# Clinical and Demographic Factors, Treatment Patterns, and Overall Survival Associated With Rare Triple-Negative Breast Carcinomas in the US

**DOI:** 10.1001/jamanetworkopen.2021.4123

**Published:** 2021-04-12

**Authors:** Elizabeth B. Elimimian, Thomas A. Samuel, Hong Liang, Leah Elson, Nadeem Bilani, Zeina A. Nahleh

**Affiliations:** 1 Department of Hematology-Oncology, Maroone Cancer Center, Cleveland Clinic Florida, Weston, Florida

## Abstract

**Importance:**

Triple-negative breast cancers are known collectively to demonstrate a more aggressive clinical course and earlier recurrence than cancers of other histological subtypes. However, the literature on rare triple-negative breast cancers and the association of histological type with survival and risk of metastasis is sparse.

**Objective:**

To present the clinical and demographic characteristics, treatment patterns, and overall survival (OS) for histologically rare (<10% of breast cancers) triple-negative breast cancer types: medullary carcinoma, adenoid cystic carcinoma, and metaplastic breast carcinoma.

**Design, Setting, and Participants:**

This cohort study was performed in the US using data reported by the National Cancer Database between 2010 and 2016. Confirmed cases of medullary carcinoma, adenoid cystic carcinoma, and metaplastic breast cancer were analyzed. Univariable analyses and multivariable Cox regression models were performed. Data analysis was performed from April to May 2020.

**Main Outcomes and Measures:**

The primary outcome was 5-year OS. Secondary outcomes included site of metastasis, effect of immunohistochemistry, management, and 2-year mortality.

**Results:**

A total of 8479 patients with breast cancer (mean [SD] age; 62.6 [14.3] years; 8435 women [99.48%]) were analyzed. Metaplastic carcinoma was the most commonly diagnosed histological type in this cohort, with 6867 patients (81%), followed by 1357 (16%) with adenoid cystic carcinoma and only 255 (3%) with medullary carcinoma. Medullary carcinoma presented earlier in life, at a median (interquartile range) age of 53 (45-62) years, compared with 62 (53-72) years for patients with adenoid cystic carcinoma and 63 (52-74) years for patients with metaplastic carcinoma. The proportion of tumors with triple-negative immunohistochemistry varied by histological type for medullary carcinoma (57 patients [22.4%]), adenoid cystic carcinoma (653 patients [48.1%]), and metaplastic carcinoma (3637 patients [53.0%]). Patients with adenoid cystic carcinoma were less likely to receive radiotherapy (711 patients [52.4%]) and chemotherapy (175 patients [12.9%]) compared with patients with medullary carcinoma (radiotherapy, 156 patients [61.2%]; chemotherapy, 190 patients [74.5%]) and metaplastic carcinoma (radiotherapy, 3416 patients [49.7%]; chemotherapy, 4709 patients [68.6%]). The 5-year OS rate was superior for patients with medullary (91.7%) and adenoid cystic carcinoma (88.4%) compared with patients with metaplastic carcinoma (63.1%). The 5-year mortality rate for adenoid cystic carcinoma was 8.33% vs 36.91% for metaplastic carcinoma.

**Conclusions and Relevance:**

Nationally, over the course of 7 years, medullary carcinoma was most common and metaplastic carcinoma had the worst 5-year OS among the rare histological breast cancer subtypes analyzed. Factors associated with a poor prognosis for metaplastic carcinoma included advanced stage, lung metastasis, older age, and not receiving chemotherapy or radiation therapy. Future research focusing on rare subtypes of breast cancer is desirable and could inform the optimal management of these relatively understudied carcinomas.

## Key Points

**Question:**

Do all triple-negative breast cancers demonstrate similar aggressiveness and response to treatment or does heterogeneity exist among triple-negative breast cancers secondary to histological type?

**Findings:**

In this cohort study of 8479 patients with rare breast cancers, adenoid cystic carcinomas and metaplastic breast cancer had similar proportions of tumors that were triple negative (48.1% vs 53.0%), but the 5-year mortality rate for adenoid cystic carcinoma was 8.33% vs 36.91% for metaplastic carcinoma.

**Meaning:**

These findings suggest that breast cancer histological type may be associated with overall survival, treatment response, and clinical course despite triple-negative receptor status.

## Teaser

This cohort study examines the clinical and demographic characteristics, treatment patterns, and overall survival of histologically rare triple-negative breast cancers, including medullary carcinoma, adenoid cystic carcinoma, and metaplastic breast carcinoma.

## Introduction

Although breast cancer accounts for approximately 25% of all female cancers worldwide and 27% of cancers in developed countries,^1^ it is the most common cancer in women globally and the second leading cause of cancer death among women in the US.^2^ Invasive ductal carcinoma is the most common breast cancer histological subtype, accounting for 55% of all breast cancer cases.^3^

Rare breast cancers with triple-negative immunohistochemistry account for approximately 15% of all invasive breast cancers^4,5^ and is defined as lacking the expression of estrogen receptor (ER), progesterone receptor (PR), and *ERBB2* (formerly *HER2*) by the 14th St Gallen International Expert Consensus.^6^ Compared with other breast cancer phenotypes, triple-negative breast cancers demonstrate an aggressive clinical course, and patients with such cancers exhibit earlier recurrence (1-3 years after diagnosis), with most deaths occurring within the first 5 years following treatment. Patients with triple-negative breast cancer have poor outcomes because they are not managed with targeted therapies such as endocrine therapy or *ERBB2* receptor antagonists.^7^ Consequently, chemotherapy is currently the mainstay systemic therapy for triple-negative breast cancer. However, patients with triple-negative breast cancer who are treated with chemotherapy might exhibit worse outcomes than patients with other breast cancer subtypes.^8,9^ More clinically efficacious targeted therapies for triple-negative breast cancer are still being investigated; anti–programmed death ligand–1 (PD-1)/PD-L1 agents are an emerging treatment modality for this breast cancer subtype and have demonstrated encouraging results.^10^

Although triple-negative breast cancers are medically managed as a single group, clinicopathologic heterogeneity exists among them. Gene expression analysis via DNA microarrays has allowed for the identification of intrinsic breast cancer subtypes such as basal-like breast cancer.^11^ Approximately 80% of triple-negative breast cancers are classified as basal-like.^12^ Claudin-low tumors are another molecular subtype of triple-negative breast cancer, with interferon-enriched cells that have properties similar to those of stems cells.^7^ These tumors have a better prognosis than other triple-negative breast cancers.^7^ Although there are recognized molecular, histological, and genetic differences among triple-negative breast cancers, the first-line treatment of all triple-negative breast cancer continues to be chemotherapy; this often leads to chemotherapy resistance.^13,14^

Rare histological subtype breast cancers comprise less than 10% of breast cancers.^15^ Although the literature and research on rare histological subtype breast cancer are sparse, a more thorough understanding of the extent of the clinicopathological heterogeneity within this subtype may help to direct future research for treatment specificity. Our analysis sought to investigate the degree of heterogeneity among triple-negative breast cancers by analyzing their clinical, pathological, and molecular features. Analysis of the National Cancer Database (NCDB) over the course of 7 years provides a large representative patient population for a thorough investigation of these rare triple-negative breast cancers. We sought to describe the clinical presentation and management of triple-negative breast cancers, analyze overall survival (OS), and investigate the association of the triple-negative histological subtype with metastasis for medullary carcinoma, adenoid cystic carcinoma, and metaplastic breast carcinoma.

## Methods

Ethical approval for this cohort study was obtained from the Cleveland Clinic institutional review board. All patient data were strictly deidentified and provided, with approval from the American College of Surgeons, as part of the NCDB. No individual person’s data were included; all data are reported in an aggregate manner. Thus, informed consent was not required or sought. This study follows the Strengthening the Reporting of Observational Studies in Epidemiology (STROBE) reporting guideline.

We used an open cohort study design to evaluate rare triple-negative breast cancers in the US from 2010 through 2016 as reported by the NCDB. The NCDB is a registry that stores more than 70% of the cancer cases in the US. The information reported to the NCDB is collected from more than 1500 Commission on Cancer–accredited medical institutions.^16^ Data fidelity is ensured via standardized reporting measures, specified by Facility Oncology Registry Data Standards, the North American Association of Central Cancer Registries,^17^ and the American College of Surgeons. Completeness of data is assessed annually before release. One patient had a missing follow-up time and was excluded from any survival analysis.

The study size is reflective of cases meeting eligibility criteria based on histological diagnosis, year of diagnosis, and staging. NCDB cases diagnosed with a histologically rare breast carcinoma of triple-negative immunohistochemistry were eligible. Rare histological subtype was defined as comprising less than 10% of NCDB breast carcinomas. Cases of medullary carcinoma, adenoid cystic carcinoma, or metaplastic carcinoma, as determined by the *International Classification of Disease for Oncology* (*ICD-O3*) between 2010 and 2016, were included for final analysis (*ICD-O3* codes 8512, 8513, 8480, and 8200). Cases diagnosed before 2010 were excluded because *ERBB2* status was not widely collected until after 2009. Medullary carcinoma includes histological diagnosis of medullary carcinoma with lymphoid stroma and atypical medullary carcinoma. All breast cancers were staged using the 7th edition of the American Joint Committee on Cancer staging manual. Stages III and IV were combined to increase statistical power. Cases with an unknown stage at diagnosis were excluded.

### Statistical Analysis

We performed univariable analyses to compare the clinical characteristics and mortality among subtypes. The χ^2^ test and Wilcoxon rank-sum test were used for categorical variables and continuous variables, respectively, and odds ratios (ORs) were calculated to determine whether a triple-negative breast cancer would be associated with increased risk of metastasis for each subtype. Follow-up time was date of diagnosis to date of death from any cause or last alive contact; patients still alive were censored for OS.

Multivariable Cox regression models, with backward elimination method, were used to identify the most important factors associated with OS, in which significant factors being identified by univariable Cox model were used as explanatory variables in the multivariable Cox regression models. The model for medullary carcinoma included age, insurance, and cancer stage; age was eliminated by backward elimination. The model for adenoid cystic carcinoma included age, insurance, stage, Charlson Comorbidity Index score, tumor grade, lymph node status, hormone therapy, radiation therapy, primary site surgery, bone metastasis, and lung metastasis; insurance, primary site surgery, bone metastasis, and lung metastasis were eliminated by backward elimination. The model for metaplastic breast cancer included age, insurance, cancer stage, Charlson Comorbidity Index score, tumor grade, lymph node status, income, chemotherapy, hormone therapy, radiation therapy, primary site surgery, liver metastasis, brain metastasis, bone metastasis, and lung metastasis; brain metastasis was eliminated by backward elimination.

The Kaplan-Meier method was used to estimate OS, and the log-rank test was used to compare triple-negative breast cancer histological subtypes. Overall mortality was defined as death from any cause. Two-year and 5-year mortality were derived from product-limit survival estimate, and the *P* values were derived from log-rank test. Patient characteristics, 30-day mortality, 90-day mortality, 2-year mortality, and 5-year mortality were summarized using median and interquartile range (IQR) for continuous variables and frequency and percentage for categorical variables. Significance levels were interpreted as 2-sided *P* < .05. All statistical analyses were conducted with SAS statistical software version 9.4 (SAS Institute). Data analysis was performed from April to May 2020.

## Results

A total of 8479 patients with breast cancer (mean [SD] age; 62.6 [14.3] years; 8435 women [99.48%]) were included in this analysis. Mean (SD) follow-up intervals were 81.0 (42.7) months for medullary carcinoma, 55.6 (36.5) months for adenoid cystic carcinoma, and 44.7 (34.8) months for metaplastic carcinoma. Univariable analyses (Table 1) revealed that most cases had a histological diagnosis of metaplastic carcinoma (6867 patients [81%]), followed by adenoid cystic carcinoma (1357 patients [16%]) and medullary carcinoma (255 patients [3%]). Medullary carcinoma had the lowest median (IQR) age at diagnosis (53 [45-62] years) compared with adenoid cystic carcinoma (62 [53-72] years) and metaplastic carcinoma (63 [52-74] years). Non-Hispanic White patients were the largest racial/ethnic group with medullary carcinomas (189 patients [74.4%]), adenoid cystic carcinomas (1125 patients [84%]), and metaplastic carcinomas (5407 patients [79.3%]). The majority of patients with all 3 rare triple-negative breast cancer subtypes at the time of diagnosis had no comorbidities (medullary carcinoma, 219 patients [85.9%]; adenoid cystic carcinoma, 1175 patients [86.0%]; metaplastic carcinoma, 5435 patients [79.2%]). Most patients with medullary carcinoma and adenoid cystic carcinoma had private insurance (164 patients [64.3%] and 688 patients [50.7%], respectively), whereas among patients with metaplastic carcinoma, 3055 (44.5%) had private insurance and 2951 (43.0%) had Medicare insurance.

**Table 1.** Univariable Analysis of Baseline Characteristics

Variable	Patients, No. (%)			* P* value
	Medullary carcinoma (n = 255)	Adenoid cystic carcinoma (n = 1357)	Metaplastic breast cancer (n = 6867)	
Age, median (IQR), y	53 (45-62)	62 (53-72)	63 (52-74)	<.001
Age, y				
<60	178 (69.8)	579 (42.7)	2825 (41.1)	<.001
≥60	77 (30.2)	778 (57.3)	4042 (58.9)	
Comorbidities, No.				
0	219 (85.9)	1175 (86.0)	5435 (79.2)	<.001
1	32 (12.5)	148 (11)	1059 (15.4)	
≥2	4 (1.6)	34 (3)	373 (5.4)	
Race				
White	189 (74.4)	1125 (84)	5407 (79.3)	<.001
Black	56 (22.1)	159 (12)	1163 (17.0)	
Other^a^	9 (3.5)	58 (4)	253 (3.7)	
Insurance status				
Not insured	7 (2.7)	29 (2.1)	147 (2.1)	<.001
Private insurance	164 (64.3)	688 (50.7)	3055 (44.5)	
Medicaid	28 (11.0)	78 (5.8)	578 (8.4)	
Medicare	51 (20.0)	540 (39.8)	2951 (43.0)	
Unknown	5 (2.0)	22 (1.6)	136 (2.0)	
Annual household income, $				
<40 227	64 (25.1)	221 (16.3)	1234 (18.0)	<.001
40 227-50 353	66 (25.9)	257 (18.9)	1467 (21.4)	
50 354-63 332	54 (21.2)	335 (24.7)	1548 (22.5)	
≥63 333	70 (27.4)	531 (39.1)	2534 (36.9)	
Unknown	1 (0.4)	13 (1.0)	84 (1.2)	
Grade				
Well	3 (1.2)	520 (38.3)	140 (2.0)	<.001
Moderately	13 (5.1)	320 (23.6)	790 (11.5)	
Poorly	210 (82.4)	138 (10.2)	4811 (70.1)	
Undifferentiated	5 (2.0)	7 (0.5)	147 (2.1)	
Unknown	24 (9.4)	372 (27.4)	979 (14.3)	
Lymph nodes involved, No.				
0	191 (74.9)	1049 (77)	4589 (66.8)	<.001
1	26 (10.2)	26 (2)	521 (7.6)	
>1	28 (11.0)	13 (1)	670 (9.8)	
Unknown	10 (3.9)	269 (20)	1087 (15.8)	
Estrogen receptor status				
Positive or other	67 (26.3)	343 (25)	1307 (19)	<.001
Negative	188 (73.7)	1014 (75)	5560 (81)	
Progesterone receptor status				
Positive or other	46 (18.0)	239 (17.6)	967 (14.1)	<.001
Negative	209 (82.0)	1118 (82.4)	5900 (85.9)	
*ERBB2* status				
Positive	11 (4.3)	6 (0.4)	305 (4.4)	<.001
Negative	81 (31.8)	856 (63.1)	4646 (67.7)	
Other or unknown	163 (63.9)	495 (36.5)	1916 (27.9)	
Primary site surgery				
No	1 (0.4)	35 (2.6)	430 (6.2)	<.001
Yes	254 (99.6)	1319 (97.2)	6432 (93.7)	
Unknown	0	3 (0.2)	5 (0.1)	
Non–primary site surgery				
No	249 (97.7)	1343 (99.0)	6721 (97.9)	<.001
Yes	0	3 (0.2)	36 (0.5)	
Unknown	6 (2.3)	11 (0.8)	110 (1.6)	
Chemotherapy				
No	56 (22.0)	1117 (82.3)	2043 (29.7)	<.001
Yes	190 (74.5)	175 (12.9)	4709 (68.6)	
Unknown	9 (3.5)	65 (4.8)	115 (1.7)	
Hormone therapy				
No	188 (73.7)	1142 (84.2)	5695 (82.9)	<.001
Yes	54 (21.2)	152 (11.2)	902 (13.1)	
Unknown	13 (5.1)	63 (4.6)	270 (3.9)	
Immunotherapy				
No	243 (95.2)	1346 (99.2)	6642 (96.7)	<.001
Yes	6 (2.4)	3 (0.2)	190 (2.8)	
Unknown	6 (2.4)	8 (0.6)	35 (0.5)	
Radiation therapy				
No	94 (36.9)	630 (46.4)	3419 (49.8)	<.001
Yes	156 (61.2)	711 (52.4)	3416 (49.7)	
Unknown	5 (2.0)	16 (1.2)	32 (0.5)	
Stage				
0	2 (0.8)	22 (1.6)	45 (0.7)	<.001
I	119 (46.6)	904 (66.6)	1865 (27.2)	
II	118 (46.3)	408 (30.1)	3727 (54.3)	
III or IV	16 (6.3)	23 (1.7)	1230 (17.9)	<.001
Metastasis				
Bone				
No or unknown	255 (100)	1354 (99.8)	6743 (98.2)	<.001
Yes	0	3 (0.2)	124 (1.8)	
Brain				
No or unknown	255 (100)	1357 (100	6836 (99.5)	<.001
Yes	0	0	31 (0.5)	
Liver				
No or unknown	254 (99.6)	1356 (99.9)	6804 (99.1)	<.001
Yes	1 (0.4)	1 (0.1)	63 (0.9)	
Lung				
No or unknown	255 (100)	1350 (99.5)	6691 (97.4)	
Yes	0	7 (0.5)	176 (2.6)	
Mortality				
30 d	0	1 (0.09)	34 (0.61)	
90 d	0	4 (0.34)	126 (2.26)	
Mortality^b^				
2 y	6 (3.08)	40 (3.83)	1245 (22.60)	<.001
5 y	18 (8.33)	100 (11.61)	1844 (36.91)	

Abbreviation: IQR, interquartile range.

^a^ Includes Hispanic, Asian, and unknown.

^b^ Data for 2- and 5-year mortality were derived from product-limit survival estimate, and the *P* values were derived from log-rank test. Mortality was defined as the rate of death from any cause.

**Figure 1.**
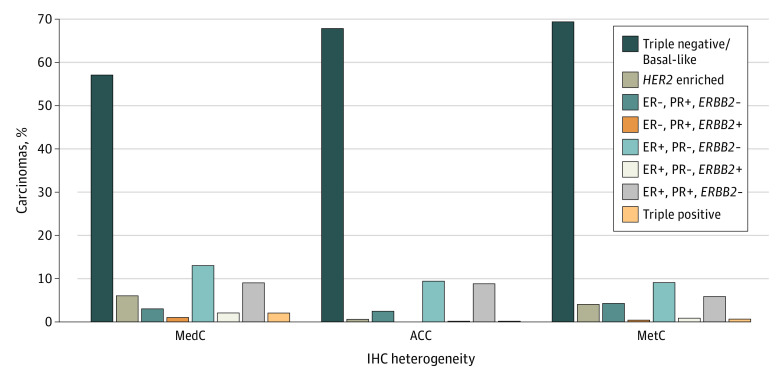
Immunohistochemistry Combinations for Medullary Carcinoma (MedC), Adenoid Cystic Carcinoma (ACC), and Metaplastic Breast Cancer (MetC) ER indicates estrogen receptor; PR, progesterone receptor.

Most medullary carcinomas (210 tumors [82.4%) and metaplastic carcinomas (4811 tumors [70.1%]) were poorly differentiated at presentation vs only 138 adenoid cystic carcinomas (10.2%). ER expression, PR expression, and *ERBB2* oncogene expression were low for cases of medullary carcinoma (67 [26.3%], 46 [18.0%], and 11 [4.3%] cases, respectively), adenoid cystic carcinoma (343 [25.3%], 239 [17.6%], and 6 [0.4%] cases, respectively), and metaplastic carcinoma (1307 [19.0%], 967 [14.1%], and 305 [4.4%] cases, respectively) (Figure 1). Metaplastic and adenoid cystic carcinomas had a similar composition of triple-negative breast cancer cells (48.1% [653 patients] vs 53.0% [3637 patients]), but that for medullary carcinoma was only 22.4% (57 patients). Metaplastic carcinoma had the highest percentage of cases with stage III and IV disease (1230 patients [17.9%]), with 1865 cases (27.2%) diagnosed at stage I and 3727 cases (54.3%) diagnosed at stage II. Metaplastic carcinoma was most likely to metastasize to the brain (31 cases [0.5%]), the liver (63 cases [0.9%]), and the lung (176 cases [2.6%]).

Surgery was performed at the primary tumor site for 254 patients with medullary carcinomas (99.6%), 1319 patients with adenoid cystic carcinomas (97.2%), and 6432 patients with metaplastic carcinomas (93.7%). Chemotherapy was given to the majority of patients with medullary carcinoma (190 patients [74.5%]) and metaplastic carcinoma (4709 patients [68.6%]), but rarely to patients with adenoid cystic carcinoma (175 patients [12.9%]). The use of hormonal therapy was low for patients with medullary carcinoma (54 patients [21.2%]), adenoid cystic carcinoma (152 patients [11.2%]), and metaplastic carcinoma (902 patients [13.1%]). Radiation therapy was given to 61.2% of patients with medullary carcinoma (156 patients), 52.4% of patients (711 patients) with adenoid cystic carcinomas, and 49.7% of those with metaplastic carcinoma (3416 patients).

Multivariable analysis (Table 2) revealed that several variables, treatments, and tumor characteristics were associated with an adverse prognosis. Having Medicare insurance (hazard ratio [HR], 5.70; 95% CI, 2.58-12.59; reference, private insurance) and stage III or stage IV disease (HR, 6.48; 95% CI, 1.87-22.37; reference, stage 0 or 1) were associated with an adverse prognosis for medullary carcinoma cases. There were no factors associated with adverse prognosis unique to adenoid cystic carcinoma cases. Income, treatment, and metastatic site were factors associated with adverse prognosis unique to metaplastic carcinoma cases. Patients with metaplastic carcinoma who had an annual household income of less than $40 227 (HR, 1.20; 95% CI, 1.06-1.35) or $50 354 to $63 332 (HR, 1.17; 95% CI, 1.04-1.32) experienced decreased survival compared with patients with incomes of $63 333 or higher. Not undergoing surgery at the primary tumor site (HR, 1.64; 95% CI, 1.38-1.94; reference, undergoing primary tumor site surgery) and not receiving chemotherapy (HR, 1.82; 95% CI, 1.65-2.01; reference, receiving chemotherapy) were associated with decreased survival among patients with metaplastic carcinomas. Metastasis to the bone (HR, 1.67; 95% CI, 1.30-2.16; reference, no bone metastasis), the liver (HR, 1.50; 95% CI, 1.07-2.09; reference, no liver metastasis), or the lung (HR, 2.63; 95% CI, 2.11-3.27; reference, no lung metastasis) was associated with decreased survival for metaplastic carcinomas.

**Table 2.** Multivariable Cox Regression Analyses for Survivorship by Histological Type^a^

Variable	Medullary carcinoma		Adenoid cystic carcinoma		Metaplastic breast cancer	
	HR (95% CI)	*P* value	HR (95% CI)	*P* value	HR (95% CI)	*P* value
Age						
<60 y	NA	NA	1 [Reference]	NA	1 [Reference]	NA
≥60 y	NA	NA	3.25 (2.13-4.95)	<.001	1.42 (1.25-1.60)	<.001
Charlson Comorbidity Index score						
0	NA	NA	1 [Reference]	NA	1 [Reference]	NA
1	NA	NA	1.70 (1.10-2.46)	.02	1.28 (1.14-1.44)	<.001
≥2	NA	NA	2.77 (1.35-5.70)	.006	1.64 (1.39-1.93)	<.001
Race						
White	1 [Reference]	NA	1 [Reference]	NA	1 [Reference]	NA
Black	NA	NA	NA	NA	NA	NA
Other^b^	NA	NA	NA	NA	NA	NA
Insurance status						
Not insured or unknown	4.10 (0.88-19.10)	.07	NA	NA	1.15 (0.92-1.44)	.23
Private insurance	1 [Reference]		NA	NA	1 [Reference]	NA
Medicaid	0.54 (0.07-4.17)	.55	NA	NA	0.99 (0.83-1.18)	.92
Medicare	5.70 (2.58-12.59)	<.001	NA	NA	1.36 (1.20-1.53)	<.001
Annual household income, $						
<40 227	NA	NA	NA	NA	1.20 (1.06-1.35)	.005
40 227-50 353	NA	NA	NA	NA	1.00 (0.89-1.13)	.95
50 354-63 332	NA	NA	NA	NA	1.17 (1.04-1.32)	.008
≥63 333	NA	NA	NA	NA	1 [Reference]	NA
Unknown	NA	NA	NA	NA	0.83 (0.55-1.26)	.38
Grade						
1	NA	NA	1 [Reference]	NA	1 [Reference]	NA
2	NA	NA	1.39 (0.87-2.21)	.16	1.20 (0.82-1.76)	.34
3-4	NA	NA	2.15 (1.33-3.46)	.002	1.45 (1.02-2.08)	.04
Unknown	NA	NA	1.20 (0.78-1.86)	.41	1.31 (0.90-1.90)	.15
Lymph nodes involved						
No	NA	NA	1 [Reference]	NA	1 [Reference]	NA
Yes	NA	NA	4.25 (2.42-7.46)	<.001	1.63 (1.43-1.85)	<.001
Unknown	NA	NA	1.94 (1.34-2.81)	<.001	1.74 (1.55-1.96)	<.001
Estrogen receptor, progesterone receptor, and *ERBB2* status						
Triple negative	NA	NA	NA	NA	NA	NA
Non–triple negative	1 [Reference]	NA	1 [Reference]	NA	1 [Reference]	NA
Primary site surgery						
No or unknown	NA	NA	NA	NA	1.64 (1.38-1.94)	<.001
Yes	NA	NA	NA	NA	1 [Reference]	NA
Non–primary site surgery						
No or unknown	NA	NA	NA	NA	NA	NA
Yes	1 [Reference]	NA	1 [Reference]	NA	1 [Reference]	NA
Chemotherapy						
No or unknown	NA	NA	NA	NA	1.82 (1.65-2.01)	<.001
Yes	NA	NA	NA	NA	1 [Reference]	NA
Hormone therapy						
No or unknown	NA	NA	2.31 (1.12-4.78)	.02	1.29 (1.12-1.49)	.001
Yes	NA	NA	1 [Reference]	NA	1 [Reference]	NA
Immunotherapy						
No or unknown	NA	NA	NA	NA	NA	NA
Yes	1 [Reference]	NA	1 [Reference]	NA	1 [Reference]	NA
Radiation therapy						
No or unknown	NA	NA	1.50 (1.05-2.15)	.03	1.42 (1.29-1.57)	<.001
Yes	NA	NA	1 [Reference]	NA	1 [Reference]	NA
Bone metastasis						
No or unknown	NA	NA	NA	NA	1 [Reference]	NA
Yes	NA	NA	NA	NA	1.67 (1.30-2.16)	<.001
Brain metastasis						
No or unknown [reference]	NA	NA	NA	NA	NA	NA
Yes	NA	NA	NA	NA	NA	NA
Liver metastasis						
No or unknown	NA	NA	NA	NA	1 [Reference]	NA
Yes					1.50 (1.07-2.09)	.02
Lung metastasis						
No or unknown	NA	NA	NA	NA	1 [Reference]	NA
Yes	1 [Reference]	NA	NA	NA	2.63 2.11-3.27)	<.001
Stage						
0 or 1 [reference]	2.41 (1.00-5.82)	.05	1 [Reference]	NA	1 [Reference]	NA
2	6.48 (1.87-22.37)	.003	1.79 (1.26-2.56)	.001	1.99 (1.74-2.26)	<.001
3 or 4	NA	NA	3.68 (1.78-7.59)	<.001	4.47 (3.82-5.22)	<.001

Abbreviations: HR, hazard ratio; NA, not applicable.

^a^ The model for medullary carcinoma included age, insurance, and stage; age was eliminated by backward elimination. The model for adenoid cystic carcinoma included age, insurance, stage, Charlson Comorbidity Index score, grade, lymph nodes, hormone therapy, radiation therapy, primary site surgery, bone metastasis, and lung metastasis; insurance, primary site surgery, bone metastasis, and lung metastasis were eliminated by backward elimination. The model for metaplastic breast cancer included age, insurance, stage, Charlson Comorbidity Index score, grade, lymph nodes, income, chemotherapy, hormone therapy, radiation therapy, primary site surgery, liver metastasis, brain metastasis, bone metastasis, and lung metastasis; brain metastasis was eliminated by backward elimination.

^b^ Includes Hispanic, Asian, and unknown.

Only metaplastic carcinomas were associated with metastasis. Triple-negative metaplastic breast carcinoma was more likely than the other histological subtypes to metastasize to the lungs (OR, 2.04, 95% CI, 1.48-3.88; *P* < .001) and the bone (OR, 1.75; 95% CI, 1.20-2.55; *P* = .003) (Table 3). The OR for metastasis could not be determined for patients with medullary carcinoma and patients with adenoid cystic carcinoma, because the event numbers were too small. In analyzing the association of triple-negative breast cancer with OS, triple-negative breast cancer was not associated with a significant decrease in OS for any the breast cancer subtypes compared with all other immunohistochemistry (IHC) combinations (eFigure in the Supplement).

**Table 3.** Association Between Triple-Negative Receptor Status and Metastasis

Type of cancer	Patients, No.			
	Bone	Brain	Liver	Lung
Medullary carcinoma (n = 255)				
Triple negative (n = 57)	0	0	1	0
Non–triple negative (n = 198)	0	0	0	0
*P* value	NA	NA	.22	NA
Adenoid cystic carcinoma (n = 1357)				
Triple negative (n = 653)	2	0	1	6
Non–triple negative (n = 704)	1	0	0	1
*P* value	.61	NA	.48	.06
Metaplastic carcinoma (n = 6867)				
Triple negative (n = 3637)	82	16	37	122
Non–triple negative (n = 3230)	42	15	26	54
*P* value	.003	.88	.36	<.001
OR (95% CI)^a^	1.75 (1.20-2.55)	0.95 (0.47-1.92)	1.27 (0.77-2.1)	2.04 (1.48-3.88)

Abbreviations: NA, not applicable; OR, odds ratio.

^a^ Triple negative vs non–triple negative.

**Figure 2.**
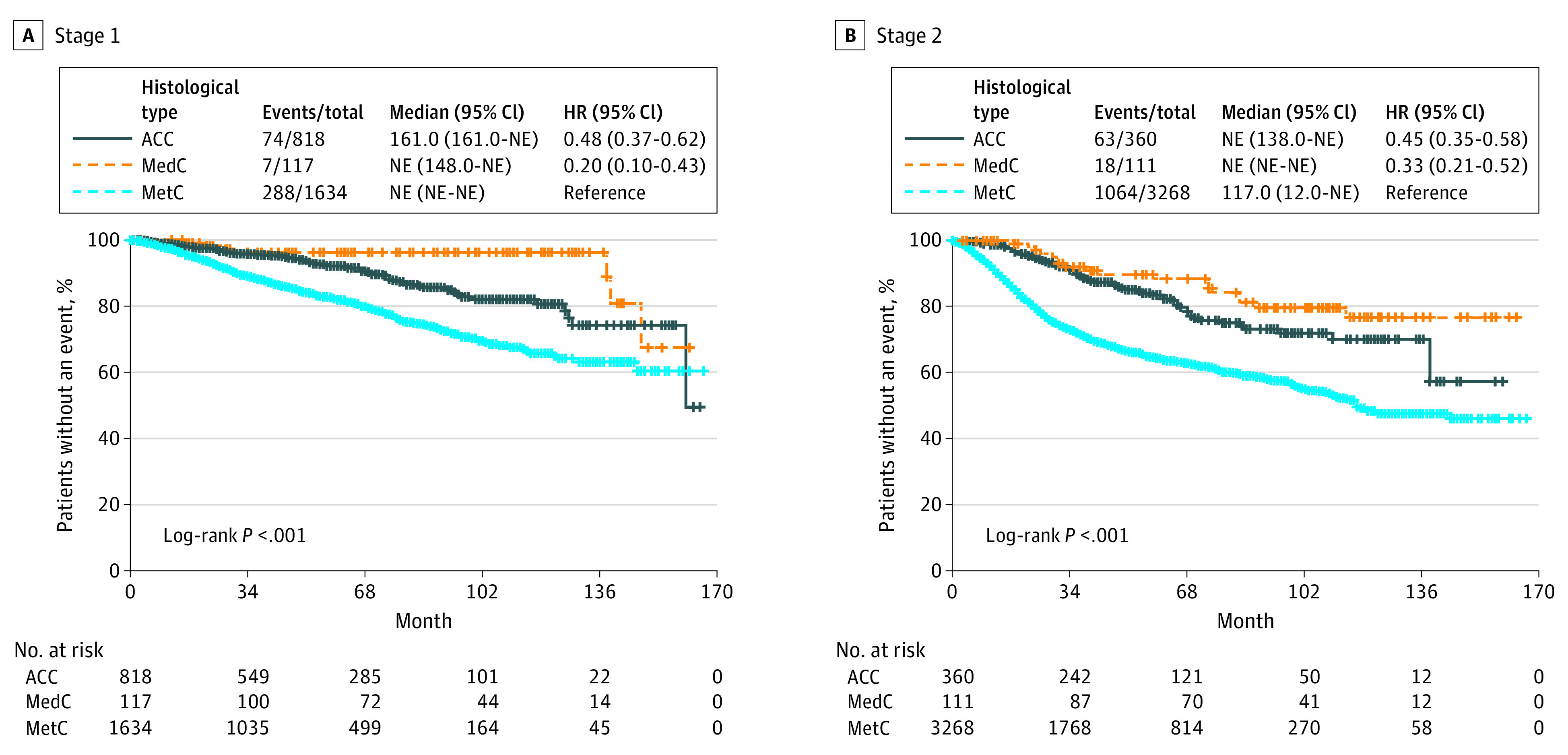
Overall Survival for Triple-Negative Breast Cancer by Stage ACC indicates adenoid cystic carcinoma; HR, hazard ratio; MedC, medullary carcinoma; MetC, metaplastic breast cancer; NE, not estimated.

The 5-year OS rate was superior for patients with medullary (91.7%) and adenoid cystic carcinoma (88.4%) compared with patients with metaplastic carcinoma (63.1%). The 5-year mortality rate was 8.33% for medullary carcinomas, 11.61% for adenoid cystic carcinomas, and 36.91% for metaplastic carcinomas. Kaplan-Meier survival analysis revealed that OS was best for patients with medullary carcinoma at all stages (Figure 2); at stages II, III, and IV disease, the OS was lowest for metaplastic carcinomas. At stages III and IV, the likelihood of death for patients with adenoid cystic carcinoma was approximately two-thirds that of patients with metaplastic carcinomas; the mortality rate for patients with medullary carcinoma was approximately 24% of the rate death recorded for patients with metaplastic carcinomas.

## Discussion

This analysis is one of the largest studies investigating and updating the demographic and clinical characteristics and OS of patients with rare triple-negative breast cancers.^18,19,20^ Most of the patients were White and presented with stage I or stage II tumors that were poorly differentiated. Patients with metaplastic breast triple-negative breast cancer were more than twice as likely to present with metastasis to the lungs and 1.75 times as likely to present with metastasis to the bone. For each rare breast cancer subtype, triple-negative IHC was not associated decreased OS compared with other IHC combinations. Some of our findings were congruent with previous publications on medullary carcinoma, adenoid cystic carcinoma, and metaplastic breast carcinoma but we report differences in metastasis, site of metastasis, and survival outcomes. These differences might be secondary to the degree in which other IHC combinations were expressed, the association between *ERBB2* expression and metastasis, variations in management, and chemotherapy resistance among triple-negative breast cancers.

Triple-negative breast cancer subtypes are commonly regarded as a single breast cancer subgroup and are treated as such, but variations exist by histological subtype and in IHC profiles that are associated with outcomes. All 3 rare breast cancer subtypes were primarily triple-negative breast cancer but varied in their expression of ER, PR, and *ERBB2* cell combinations. Medullary carcinoma had a higher percentage of *ERBB2*-enriched cells (ER negative, PR negative, and *ERBB2* positive) and triple-positive tumor cells (ER positive, PR positive, and *ERBB2* positive) compared with adenoid cystic carcinoma and metaplastic breast carcinoma (Figure 1). Although adenoid cystic carcinoma and metaplastic breast carcinoma had almost the same percentage of triple-negative breast cancer expression, adenoid cystic carcinoma had a larger percentage of tumor cells that were ER positive, PR positive, and *ERBB2* negative and less than 1% of tumor cells that were *ERBB2* enriched (Figure 1). Metaplastic breast carcinoma had the lowest expression of ERs (19.0%) and PRs (14.1%) but the highest *ERBB2* oncogene expression (4.4%) (Table 1); this high level of *ERBB2* expression may be one of the reasons metaplastic breast carcinoma was associated with an increased likelihood of metastasis to the lungs and bone. Although the mechanism is not fully understood, several studies have found that increased *ERBB2* oncogene expression is important in early malignant transformation.^21,22^ Overexpression of *ERBB2* has been found to promote early dissemination of incompletely transformed cells.^23^ *ERBB2* activation provides a selective advantage during tumor initiation and fosters invasive and metastatic phenotypes.^24,25^ Increased understanding of genomic profiles and IHC profiles can assist in estimating metastatic potential and can assist in the development of targeted therapies for all triple-negative breast cancer.

In general, triple-negative breast cancer tumors respond to chemotherapy, but molecular and genetic heterogeneity can lead to chemotherapy resistance and differences in mortality.^26,27,28^ Local-regional treatment, such as primary site surgery and combination chemotherapy, are the standard of care for triple-negative breast cancers.^26^ Primary site surgery was performed for 99.6% of patients with medullary carcinomas, 97.2% of patients with adenoid cystic carcinomas, and 93.7% of patients with metaplastic breast carcinomas. Chemotherapy was provided to 74.5% of patients with medullary carcinomas, 12.9% of patients with adenoid cystic carcinomas, and 68.6% of patients with metaplastic breast carcinomas. Although both metaplastic and adenoid cystic carcinomas had a similar proportion of triple-negative tumors, the outcome in survival varied between the 2 histological types. At 5 years, the OS was 91.7% for adenoid cystic carcinoma but only 63.1% for metaplastic breast carcinomas despite metaplastic breast carcinomas receiving chemotherapy at 4 times the rate of adenoid cystic carcinomas. The optimal treatment strategy for metaplastic breast carcinomas and triple-negative breast cancer remains unknown. Because of the histological, IHC, and genetic differences among triple-negative breast cancer, survival outcomes vary. As such, novel and targeted treatment approaches are desirable and should be considered for triple-negative breast cancer tumors that have historically been managed as a single group.

Numerous clinical trials of active targeted agents for the management of triple-negative breast cancer have experienced variable success rates, likely because of the molecular, genetic, and clinical heterogeneity of triple-negative breast cancer. Some of the more recent clinical breast cancer trials have recognized the immunogenicity of triple-negative breast cancers and have demonstrated success in managing triple-negative breast cancer with anti-PD-1/PDL1 agents.^29^ Of note, triple-negative breast cancer treated with atezolizumab (which is selective against PD-L1) achieved a greater median progression-free survival compared with triple-negative breast cancer that did not receive a targeted PD-L1 inhibitor.^30^ The success of targeted clinical trials for triple-negative breast cancer has varied. For instance, a single-group phase 2 neoadjuvant trial^31^ of cisplatin for triple-negative breast cancer achieved only a 22% pathological complete response rate, despite the fact that most of the patients had basal-like tumors. Another phase 2 study^32^ exploring cetuximab in combination with carboplatin reported a 17% response rate. Additional research focusing on triple-negative and rare breast cancer subtypes is desirable and would inform optimal management.

### Strengths and Limitations

The strengths of our analysis include the use of a large population of patients with histological breast cancers that were not commonly evaluated in literature, high fidelity of the data associated with the standardization of the reporting processes required by the NCDB, data extracted from cancer centers accredited by the American College of Surgeons, and broad scope of analysis including clinical presentation, management, and survival of patients with rare breast carcinomas. The limitations of our analysis include the use of retrospective data, limited information on the genomic profiles of the tumors and levels of claudin and Ki-67 protein (which helps control how fast cancer cells grow), and the limited reporting of *ERBB2* status before 2010.

## Conclusions

Our analysis provides outcome data, over the course of 7 years, with respect to 3 understudied rare triple-negative breast cancers. This analysis noted that triple-negative breast tumors have clinicopathological heterogeneity and variable chemotherapy resistance and variations in outcomes, with the worst 5-year OS noted for metaplastic breast carcinomas. Factors associated with poor prognosis for metaplastic breast carcinomas were advanced stage, lung metastasis, not receiving chemotherapy, and not receiving radiation therapy. Although histological subtyping of triple-negative breast cancer is not well established, our analysis suggests heterogeneity in clinical presentation, histological profile, and IHC for these 3 rare breast cancers histological subtypes, which may be of prognostic and therapeutic importance.

## Notes

Supplement. **eFigure.** The Effect of Triple Negative Receptors on OS
