# Supplementary material for: Clinical and Demographic Factors, Treatment Patterns, and Overall Survival Associated With Rare Triple-Negative Breast Carcinomas in the US
Source: JAMA Netw Open. 2021 Apr 12;4(4):e214123. doi: 10.1001/jamanetworkopen.2021.4123 (PMC8042532; doi:10.1001/jamanetworkopen.2021.4123)
Supplement: Supplement. — eFigure. The Effect of Triple Negative Receptors on OS [file jamanetwopen-e214123-s001.pdf]

Supplemental Online Content

Elimimian EB, Samuel TA, Liang H, Elson L, Bilani N, Nahleh ZA. Clinical and demographic factors, treatment patterns, and overall survival associated with rare triple-negative breast carcinomas in the US. *JAMA Netw Open*. 2021;4(4):e214123. doi:10.1001/jamanetworkopen.2021.4123

eFigure. The Effect of Triple Negative Receptors on OS

This supplemental material has been provided by the authors to give readers additional information about their work.

© 2021 Elimimian EB et al. *JAMA Network Open.*

eFigure: The Effect of Triple Negative Receptors on OS

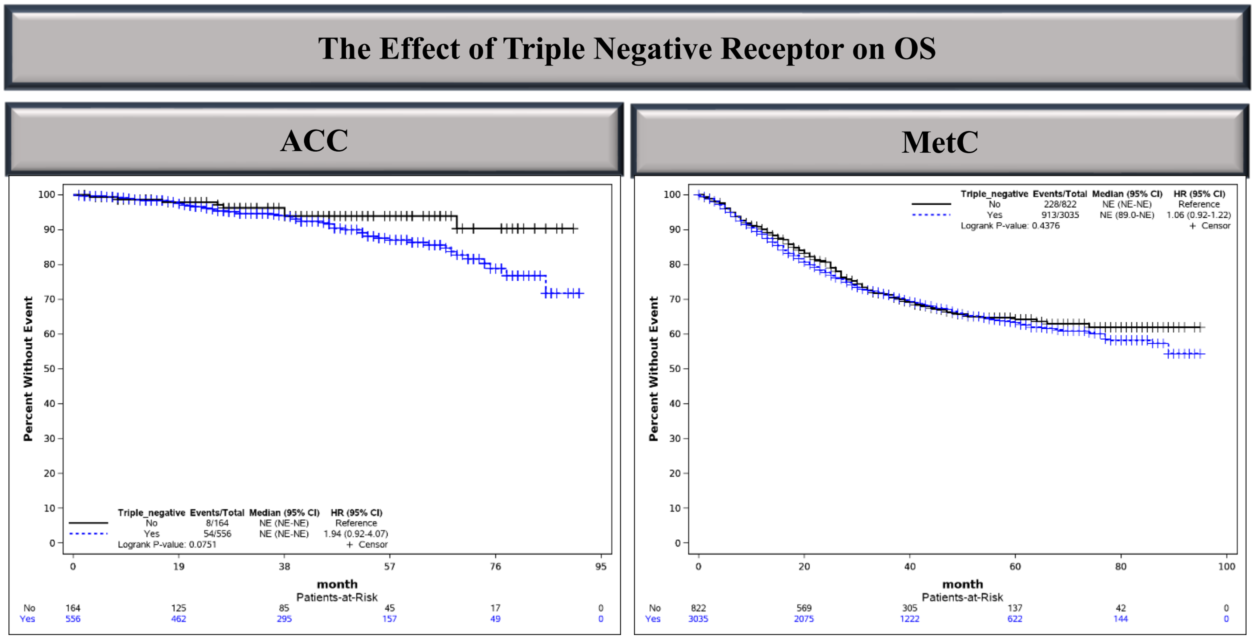

Subgroup analysis for medullary carcinoma is not shown due to sample size limitations

© 2021 Elimimian EB et al. *JAMA Network Open.*
